# Chirality Emergence in Thin Solid Films of Amino Acids by Polarized Light from Synchrotron Radiation and Free Electron Laser

**DOI:** 10.3390/ijms10073044

**Published:** 2009-07-07

**Authors:** Jun-ichi Takahashi, Hiroyuki Shinojima, Michiko Seyama, Yuko Ueno, Takeo Kaneko, Kensei Kobayashi, Hajime Mita, Mashahiro Adachi, Masahito Hosaka, Masahiro Katoh

**Affiliations:** 1 NTT Microsystem Integration Laboratories / 3-1, Morinosato-Wakamiya, Atsugi, 243-0198 Japan; E-Mails: sinojima@aecl.ntt.co.jp (H.S.); michiko@aecl.ntt.co.jp (M.S.); ueno.yuko@lab.ntt.co.jp (Y.U.); 2 Yokohama National University / 79-5, Tokiwadai, Hodogaya-ku, Yokohama, 240-8501 Japan; E-Mails: t-kaneko@ynu.ac.jp (T.K.); kkensei@ynu.ac.jp (K.K.); 3 Fukuoka Institute of Technology / 3-30-1, Wajiro-Higashi, Higashi-ku, Fukuoka, 811-0295 Japan; E-Mail: mita@fit.ac.jp (H.M.); 4 UVSOR / 38, Nishigou-Naka, Myodaiji, Okazaki, 444-8585 Japan; E-Mails: adachi@ims.ac.jp (M.A.); mkatoh@ims.ac.jp (M.K.); 5 Nagoya University / Furou-cho, Chigusa-ku, Nagoya, 464-8601 Japan; E-Mail: m-hosaka@nucl.nagoya-u.ac.jp (M.H.)

**Keywords:** chirality, circularly polarized light, amino acids, solid films, synchrotron radiation, free electron laser, origin of terrestrial homochirality

## Abstract

One of the most attractive hypothesis for the origin of homochirality in terrestrial bioorganic compounds is that a kind of “chiral impulse” as an asymmetric excitation source induced asymmetric reactions on the surfaces of such materials such as meteorites or interstellar dusts prior to the existence of terrestrial life (Cosmic Scenario). To experimentally introduce chiral structure into racemic films of amino acids (alanine, phenylalanine, isovaline, etc.), we irradiated them with linearly polarized light (LPL) from synchrotron radiation and circularly polarized light (CPL) from a free electron laser. After the irradiation, we evaluated optical anisotropy by measuring the circular dichroism (CD) spectra and verified that new Cotton peaks appeared at almost the same peak position as those of the corresponding non-racemic amino acid films. With LPL irradiation, two-dimensional anisotropic structure expressed as linear dichroism and/or linear birefringence was introduced into the racemic films. With CPL irradiation, the signs of the Cotton peaks exhibit symmetrical structure corresponding to the direction of CPL rotation. This indicates that some kinds of chiral structure were introduced into the racemic film. The CD spectra after CPL irradiation suggest the chiral structure should be derived from not only preferential photolysis but also from photolysis-induced molecular structural change. These results suggest that circularly polarized light sources in space could be associated with the origin of terrestrial homochirality; that is, they would be effective asymmetric exciting sources introducing chiral structures into bio-organic molecules or complex organic compounds.

## 1. Introduction

The origin of homochirality in terrestrial bioorganic compounds (l-amino acid, d-sugar dominant) is one of the most mysterious issues that remain unresolved in the study of chemical evolution prior to the origin of terrestrial life. Because bioorganic compounds synthesized in abiotic circumstances are intrinsically racemic mixtures of equal amounts of l- and d-bodies, it is hypothesized that chiral products originated from asymmetric chemical reactions induced by a “chiral impulse”. These types of asymmetric reactions could have possibly been derived from physically asymmetric excitation sources in space and the chiral products would have been transported to primitive Earth resulting in terrestrial homochirality (Cosmic Scenario) [1–3]. In space, asymmetric excitation would be mainly effectuated by polarized photons of synchrotron radiation (SR) from kinetic electrons captured by strong magnetic field around neutron stars or white dwarfs in supernova explosion area [1,4], or by polarized photons scattered by interstellar dust clouds in star formation areas [5,6]. Another proposed hypothesis is that polarized electrons and positrons or neutrinos produced from neutrons released in supernova explosions can cause chiral asymmetry in molecules in interstellar gas-dust clouds [7,8]. The polarized electrons are derived from parity-violating radioactive β-decay involving weak nuclear interaction (n → p^+^ + e^−^ + *ν̄*_e_). Recently, a specific physical mechanism of a relativistic neutron fireball, a relativistic chiral electron–proton plasma, has been advocated, in which the electrons carrying their helicities to the cloud show high chiral efficiency [9]. Eventually, several terrestrial observations of polarized photon radiation from space due to scattering by interstellar dust clouds in star formation areas have been reported [5,6] and ground-level or satellite observation projects are in progress.

Several experiments have already examined asymmetric photochemical reactions in simple biochemical molecules by using circularly-polarized light (CPL) [10]. In particular, asymmetric photolysis of racemized amino acids in aqueous solution by using CPL from SR, Takano *et al.* have reported asymmetric photolysis of racemic isovaline in aqueous solution [11] and Nishino *et al.* have reported pH dependence of leucine asymmetric photolysis [12]. In these experiments, they realized the simple principle that one optical isomer in optically-active compounds can be selectively-photolyzed depending on its circular dichroism (CD) in the wavelength region of the CPL. Furthermore, Takano *et al.* have recently reported CPL irradiation of aqueous solution of complex organic compounds of high molecular weight, which involves amino-acid precursors [13]. The complex organic compounds, whose molecular weight was estimated as several thousands, were produced from a gaseous mixture of molecules identified in interstellar media (comets or meteorites) by irradiation with a 3 MeV proton beam. After CPL irradiation on the amino-acid precursor products and acid hydrolysis, positive and negative enantiomeric excesses in alanine were successfully detected according to the rotation direction for right- and left-handed CPL, respectively.

On the other hand, bioorganic molecules in the solid phase contribute more than those in the liquid-phase for constructing and verifying reaction models of on-surface or surface-catalytic reactions on space materials such as interstellar dust. Meierhenrich *et al.* [14] and Tanaka *et al.* [15] have reported solid-phase leucine photolysis and Takahashi [16] also has reported solid-phase phenylalanine photolysis using SR in the vacuum ultraviolet (VUV) region.

In this paper, results of verification experiments in the laboratory are shown in order to clarify the relationship between biological chirality and asymmetric radiation as an excitation source. In this study, as the starting material for anisotropy introduction, we chose solid-phase films of optically active amino acids, - alanine (α-hydrogen amino acid), phenylalanine and tryptophan (aromatic α-hydrogen amino acids), and isovaline (α-methyl amino acid) - deposited on glass surfaces. These kinds of material systems adequately simulate molecular compounds adsorbed on low-temperature interstellar media. Aromatic hydrocarbons abundantly exist in interstellar space and play a major role related to the origin of terrestrial life [17]. It is consequently suggested that aromatic amino acids contribute significantly to the structure of the building blocks of terrestrial life. From the viewpoint of optical properties, it is well known that ultraviolet (UV) region CD spectra of aromatic amino acids in aqueous solution show clear sign inversion of the Cotton effect derived from aromatic groups, i.e., benzene or indole ring. Therefore, these CD spectroscopic characteristics not only facilitate the assignment of spectral peaks for electronic transitions but also the analysis of molecular conformation change due to surrounding circumstances. On the other hand, in amino acids in the Murchison meteorite, l-enantiomeric excess was observed in isovaline (a kind of α-methyl amino acid) [18]. Generally, the racemization rate of α-methyl amino acids is smaller than those of alpha-hydrogen amino acids. The observed enantiomeric excess of α-methyl amino acids in meteorites suggests that asymmetric reactions on surfaces of space materials really occurred and the consequent enantiomeric excess was preserved without racemization.

To clarify the experimental emergence of optical anisotropy in a solid phase experimentally, we measured the CD of amino acid films after they had been excited by polarized light. Our experiment focused on the consistency of the emerged optical anisotropy with the polarity of the polarized light. The primary goals of this work are to introduce axial-symmetric optical anisotropy by means of linearly polarized light (LPL) irradiation and to introduce mirror-symmetric anisotropy, that is “chirality” by means of left- and right-handed CPL irradiation. The emergence of chirality in bioorganic molecules in the solid phase after irradiation with CPL is effective enough to demonstrate asymmetric reactions on the surfaces of space materials, which will reveal the origins of terrestrial homochirality.

## 2. Results and Discussion

### 2.1. Polarized Light Irradiation and CD Measurement Apparatus

To introduce optical asymmetry into the racemic film deposited from dl-amino acids, we irradiated them with LPL introduced from synchrotron radiation (SR) from the normal-conducting accelerator ring (NAR) of SR facilities of Nippon Telegraph and Telephone Co. (NTT), Atsugi, Japan [19], and with CPL introduced from the free electron laser (FEL) of the UVSOR-II ring of Institute for Molecular Science (IMS), Okazaki, Japan [20],. The wavelength region of LPL was longer than 195 nm continuum, which is limited due to optical transmittance of fuzed quartz window cutting off the shorter wavelength of white SR light. On the other hand, the wavelength region of CPL was 215–216 nm monochromatic, which is tuned by the resonator condition of the FEL. The polarization purity of the CPL of the FEL was observed as above 98 % [21].

**Figure 1.**
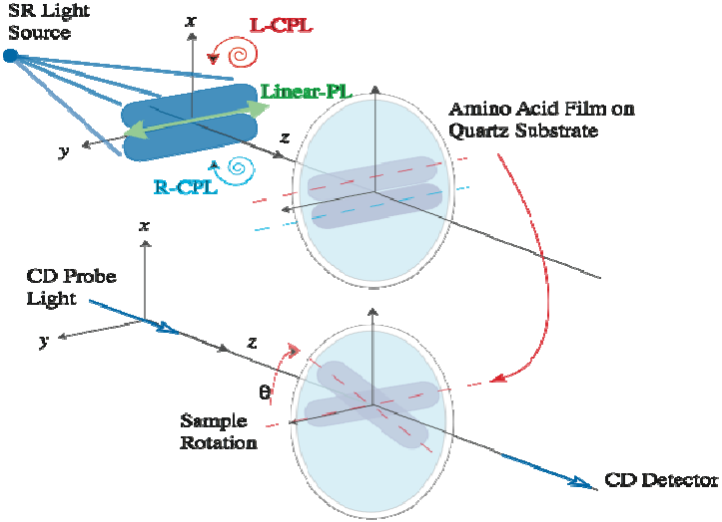
Schematic view of the optical arrangement of the light sources and sample for LPL and CPL irradiation and for CD measurement.

In order to clarify the optical anisotropy of the amino acid films, we measured the CD and photo absorption spectra of the deposited films using a commercial CD spectrometer (JASCO J-725). Figure 1 shows a schematic view of the optical arrangement of the light source and sample for LPL and CPL irradiation and for CD measurement. The sample can be rotated around the optical axis of the spectrometer by rotation angle θ (defined as the angle between the optical axis of a spectrometer and the direction of the electric field vibration of irradiated LPL on the sample).

### 2.2. Results of LPL Irradiation

#### 2.2.1. Phenylalanine

**Figure 2.**
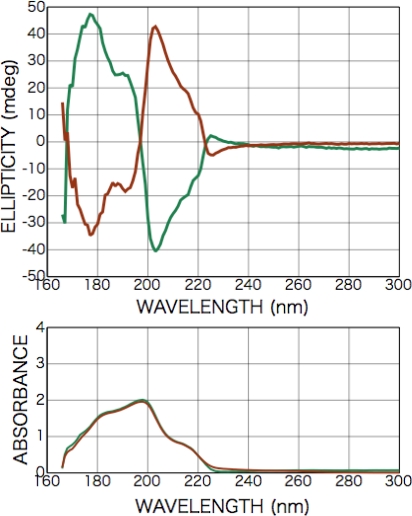
CD and photo absorption spectra of as-deposited d- (brown) and l-phenylalanine (green) films (200-nm in thickness).

**Figure 3.**
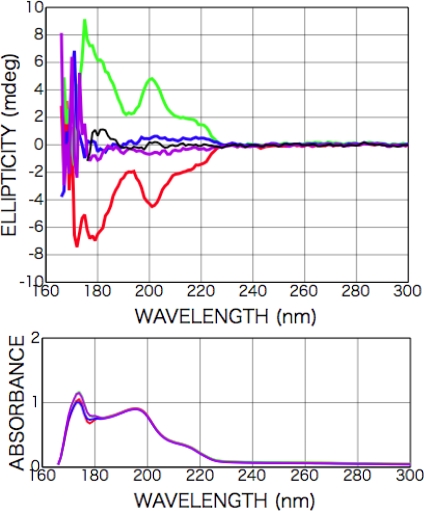
CD and photo absorption spectra of dl-phenylalanine film (100-nm in thickness); as deposited (black) and after LPL irradiation (cloured). The sample rotation angle θ = 82 deg. (red), θ = 127 deg. (purple), θ = 142 deg. (blue), and θ = 187 deg. (green).

Figure 2 shows CD and photo absorption spectra of as-deposited d- and l-phenylalanine films. Cotton peaks appeared in the CD spectra at 226 nm [carboxyl group (n-π*) and aromatic ring (π-π*)], 204 nm [aromatic ring (π-π*)], and 188 nm [higher level of carboxyl group (π-π*)]. Figure 3 shows CD and photo absorption spectra of as-deposited dl-phenylalanine film. As shown in the figure, no peaks appeared in the CD spectra because of the cancellation due to the summation of symmetric CD spectra of the d- and l-bodies.

**Figure 4.**
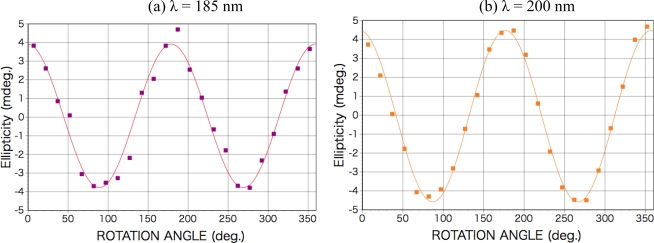
Sample rotation angle θ dependence of CD peak intensity for dl-phenylalanine film after LPL irradiation. (a) CD wavelength of 185 nm; (b) CD wavelength of 200 nm.

After the irradiation to dl-phenylalanine film with the LPL-SR (wavelength > 195 nm, total dose [storage ring current x time] 45 A min), Cotton peaks appeared in the CD spectra at almost the same peak positions as those of d- or l-amino acid films, but the signs of the peaks were all the same at each θ (Figure 3). The intensity and the sign of the peaks changed with the sample rotation angle θ. As shown in Figures 4(a) and (b), the peak intensity on the sample rotation angle changed as cos 2θ dependence at both CD wavelengths of 185 and 200 nm.

It must be noted that CD data of absorbing samples (absorbance > 1.0) should be carefully treated because of artifacts due to the interaction with CD measurement system [22]. The absorbance of the samples we used in this study ranges from 1.0 to at most 2.0 in the shorter wavelength than 210 nm. In this study, we are sure that our data were not so seriously affected by these kinds of artifacts.

Optical anisotropy measurements of solid samples using conventional CD spectrometer is in general much more difficult than those of liquid samples because of the artifacts due to the interaction between instrumental characteristics of spectrometer and linear anisotropy of solid samples, that is, linear dichroism and linear birefringence.

Shindo *et al.* [23] and Kuroda *et al.* [24] have developed a universal method for optical anisotropy measurement of solid state samples, which enables simultaneous and artifact-free determination of optical anisotropy factors; linear dichroism (LD), linear birefringence (LB), circular dichroism (CD), and circular birefringence (CB). According to their reports, the dependence of appearance CD signal (app CD) on sample rotation angle θ in case of 50-kHz modulation frequency should be summarized to the expression as below:

(1) app CD∝(Px2+Py2) [CD+½ (LD' LB−LD LB')+(−LDcos2θ+LD'sin2θ)sinα]+(Px2−Py2) [sin2a (−LBcos2θ+LB'sin2θ)+cos2a (−LBsin2θ+LB'cos2θ)]+f1 (LD,LD',LB,LB') sin4θsinα+f2 (LD,LD',LB,LB')cos4θsinα

where

LD, LD’: linear dichroism in [0° – 90°, 45° – 135°] direction
LB, LB’: linear birefringence in [0°–90°, 45°–135°] direction
P_x_^2^, P_y_^2^: transmittance of photodetector (P_x_ ^2^ / P_y_ ^2^ ≅ 1.4)
a: optical axis of photodetector (sin 2a ≅ 1)
α: residual static birefringence of modulator
f_1_, f_2_: quadratic functions of LD, LD’, LB, and LB’

As for our CD spectrometer, the ratio of P_x_ ^2^ and P_y_ ^2^ was measured by using linear polarizer as P_x_ ^2^ / P_y_ ^2^ ≅ 1.4, resulting that factor of (P_x_ ^2^ − P_y_ ^2^) is non-zero. Optical axis of photodetector, a, was also measured as sin 2a ≅ 1 and cos 2a ≅ 0. Concerning to residual static birefringence of modulator, α, in Kuroda‘s method [16], the modulator having minimum α was selected and they treated sin α as a negligible factor. In our case, α of our commercial spectrometer was not measured and we should treat sin α as a non-negligible factor in the following analysis. From the consideration of the symmetry of the LPL from SR irradiation system, it should be LD’ and LB’ = 0. Therefore, appearance CD signal is expressed, if 4θ dependence terms can be omitted, as below:

(2) $$\begin{array}{l} \text{app}\;\text{CD}\propto ({{\text{P}}_{\text{x}}}^{2}+{{\text{P}}_{\text{y}}}^{2})\;[\text{CD}-\text{LD}\text{cos}2\theta \text{sin}\alpha ]-({{\text{P}}_{\text{x}}}^{2}-{{\text{P}}_{\text{y}}}^{2})\;\text{LB}\text{cos}2\theta \\ +{\text{f}}_{1}\;(\text{LD},\text{LB})\text{sin}4\theta \text{sin}\alpha +{\text{f}}_{2}\;(\text{LD},\text{LB})\text{cos}4\theta \text{sin}\alpha \end{array}$$

From the comparison of this expression with clear cos 2θ dependence of the data in Figures 4 (a) and (b), it can be declared that no CD components but non-zero LD and/or LB components have appeared. It is therefore concluded that a kind of two-dimensional anisotropic structure expressed as at least LD and/or LB was introduced into the racemic films. In this stage we cannot numerically estimate the contribution of LD and LB because the unknown factor, sin α, still remains [25].

#### 2.2.2. Tryptophan

**Figure 5.**
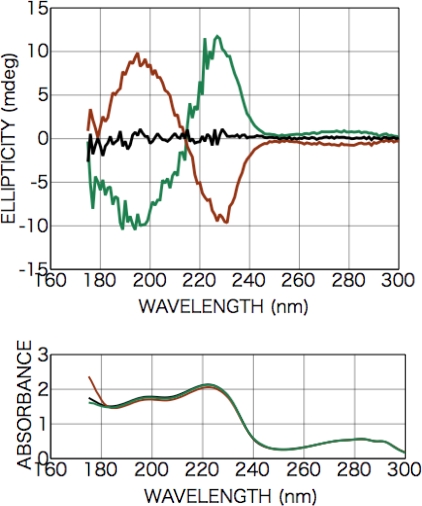
CD and photo absorption spectra of as-deposited d- (brown), l- (green), and dl-tryptophan (black) films (200-nm in thickness).

Figure 5 shows CD and photo absorption spectra of as-deposited d-, l-, and dl-tryptophan films. Two Cotton peaks appeared in the CD spectra at 228 nm [carboxyl group (n-π*)], 195 nm [aromatic ring (π-π*)] for d- and l-bodies, and no peaks appeared in the CD spectra for the dl-body, as in the same case of the dl-phenylalanine film.

**Figure 6.**
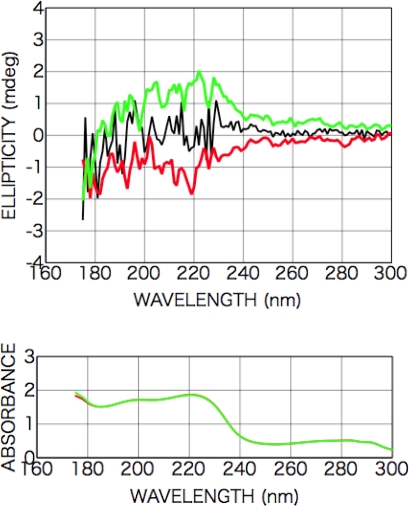
CD and photo absorption spectra of dl-tryptophan film before- (black) and after- (green and red) LPL irradiation. The sample rotation angle θ = 0 deg. (green) and θ = 90 deg. (red).

After the irradiation to dl-tryptophan film with the LPL- SR (wavelength > 195 nm, total dose [storage ring current x time] 45 A min), some new broad peaks appeared in the CD spectra at almost the same peak positions as those of corresponding non-racemic d- or l-amino acid films, but the signs of the peaks were all same at each θ as shown in Figure 6. The intensity and sign of the peaks changed with the sample rotation angle θ, and the peak intensity dependence on the sample rotation angles of 0 and 90 degree suggests cos 2θ dependence, which expresses LB and /or LD characteristics, as in the case of phenylalanine.

### 2.3. Results of CPL Irradiation

#### 2.3.1. Phenylalanine

**Figure 7.**
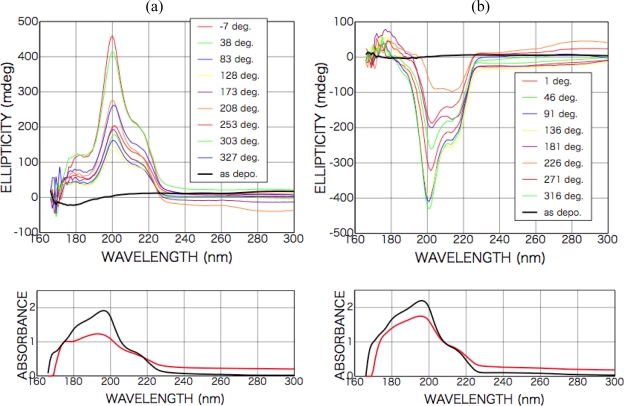
CD and photo absorption spectra of dl-phenylalanine films as-deposited (black) (200-nm in thickness) and after CPL irradiation (colored). (a) Left-handed CPL; (b) right-handed CPL. Dependence on the sample rotation angle θ is also shown.

Figure 7 shows CD and photo absorption spectra of as-deposited dl-phenylalanine films and those after irradiation with left-handed CPL (a) and right-handed CPL (b).

**Figure 8.**
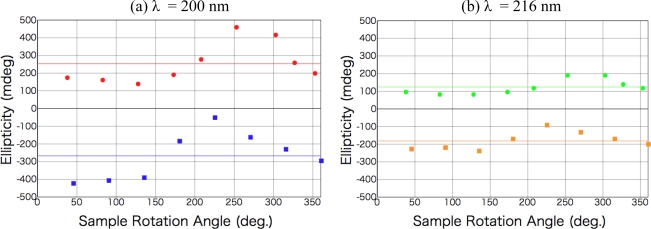
Sample rotation angle θ dependence of CD peak intensity ((a) CD wavelength 200 nm and (b) 216 nm) for dl-phenylalanine film after left-handed CPL (red for 200 nm in (a) and green for 216 nm in (b)) and right-handed CPL (blue for 200 nm in (a) and brown for 216 nm in (b)) irradiation. Straight lines show θ-independent non-zero CD components for each case.

After the irradiation with the CPL (wavelength 216 nm, total irradiation energy 6.5 mWhour), new optical anisotropy peaks appeared in the CD spectra at 216 nm [carboxyl group (n-π*) and aromatic ring (π-π*)] and 204 nm [aromatic ring (π-π*)], and the intensity of the peaks slightly changed with sample rotation angle as shown in Figures 7 (a) and (b). Figure 8 shows sample rotation angle dependence of CD peak intensity after left-handed and right-handed CPL for the CD wavelengths 200 nm and 216 nm. The signs of the peaks, however, were conserved positive with left-handed CPL irradiation and negative with right-handed CPL irradiation, independent of the sample rotation.

As shown in the expression (1), appearance CD signal has two components, that is θ-independent terms and 2θ- or 4θ-dependent terms. Figure 7 clearly shows CPL-irradiated phenylalanine film have θ-independent non-zero CD components whose signs are opposite each other corresponding with the rotation direction of left- or right-handed CPL.

This result shows that structure distortion or a conformation change of phenylalanine molecules in the film occurred due to spiral dipole polarization with intense CPL irradiation and then an optically active structure was introduced in the films. As shown in the absorption spectra, the absorbance after CPL irradiation increased on the low-energy side but decreased on the high-energy side, suggesting some molecular structure change occurred as mentioned above.

On the other hands, in the appearance CD signal additional random or 1θ-dependent-like components exist as shown in the figure. The expression (1) includes only 2θ- and 4θ-dependence components interacting with linear anisotropy, LD, LD’, LB and LB’. Furthermore, from the measurement of the symmetry of the CPL from FEL irradiation system, whose polarization purity over 98 % [21], it can be expected that LD, LD’, LB and LB’ ≅ 0. The observed 1θ-dependent-like components might be due to some kinds of material originated effects, i.e. light scattering from surfaces with random roughness or oriented micro-crystalline precipitation. Detailed material analysis using several kinds of surface analyzing methods is required in future. including the survey for the origin of much intense CD signals from CPL-irradiated phenylalanine films comparing with those from other amino acid films.

#### 2.3.2. Alanine

**Figure 9.**
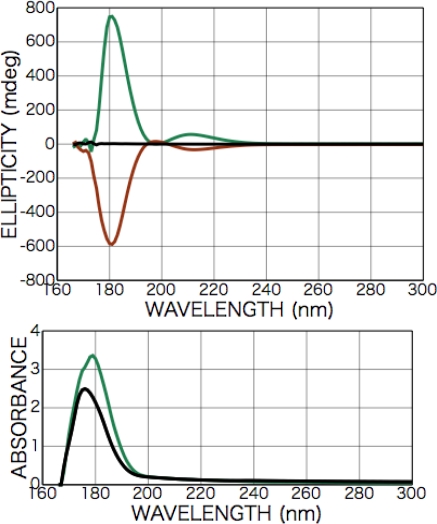
CD and photo absorption spectra of as-deposited d- (brown), l- (green), and dl-alanine (black) films (250-nm in thickness).

Figure 9 shows CD spectra of as-deposited d-, l-, and dl-alanine films. Cotton peaks appeared in the CD spectra at 210 nm [carboxyl group (n-π*)], 181 nm [carboxyl group (π-π*)] for d- and l-bodies, and no peaks appeared in the CD spectra for the dl-body, as in the case of dl-phenylalanine film (black dotted line in Figure 9).

**Figure 10.**
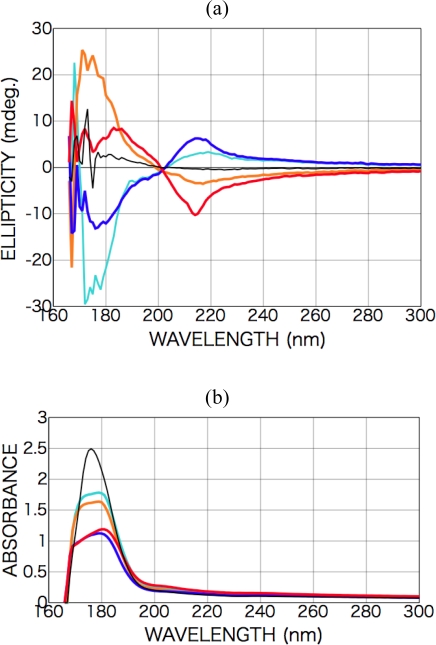
(a) CD spectra of dl-alanine films (250-nm in thickness) after CPL irradiation (colored). Left-handed CPL [orange (2 mWhour) and red (10 mWhour)]; right-handed CPL (light blue and blue). Dependence on the irradiation dose is also shown. (b) Photo absorption spectra of dl-alanine films as-deposited (black) and after CPL irradiation (colored). Left-handed CPL (orange and red); right-handed CPL [light blue (2 mWhour) and blue (10 mWhour)].

After the irradiation with the CPL (wavelength 215 nm, total irradiation energy 2 and 10 mWhour), peaks appeared in the CD spectra at 215 nm [carboxyl group (n-π*)] and 180 nm [carboxyl group (π-π*)] with an opposite sign (Figure 10 (a)). As shown in the figure, almost completely symmetric spectra were observed with left-handed CPL and right-handed CPL irradiation. The dependence on sample rotation angle was not systematically measured as phenylalanine case, but the spectra of 90-degree rotation were roughly measured. If LD and/or LB components are dominantly effective, the signs of appearance CD peaks should be inverted as shown in the expression (3). We ensured that rough spectrum profile and at least signs of two CD peaks were not changed. Therefore, observed appearance CD should be derived dominantly from chiral CD component. Furthermore, the intensity ratio of the two peaks and the intensity and the sign of the CD peaks changed with irradiation dose.

These results suggest that a chiral structure was introduced into the racemic alanine film by the CPL. As shown in the absorption spectra (Figure 10 (b)), the absorbance after CPL irradiation increased on the low-energy side but decreased on the high-energy side, suggesting some molecular structure change occurred as mentioned above as in the case of phenylalanine film.

#### 2.3.3. Isovaline

**Figure 11.**
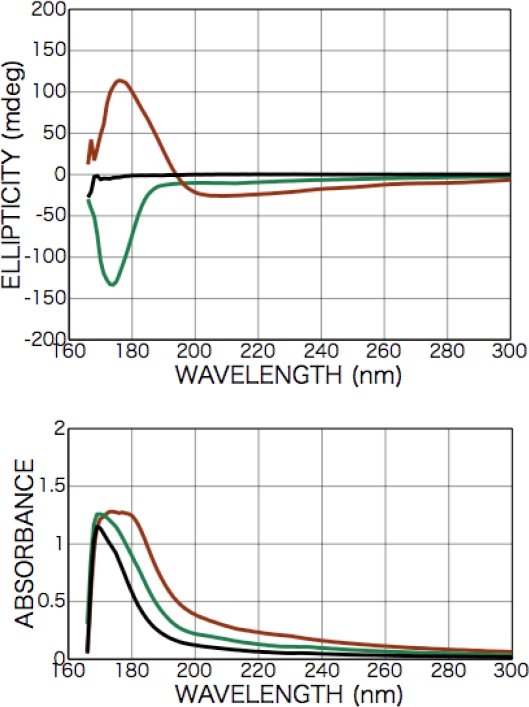
CD spectra of as-deposited d- (brown), l- (green) and dl-isovaline (black) films (100-nm in thickness).

Figure 11 shows CD spectra of as-deposited d-, l-, and dl-isovaline films. As shown in Figure 10, CD spectra of the racemic dl-isovaline film exhibited no peaks but peaks were observed in the spectra of chiral films deposited from d- or l-isovaline at 205 and 175 nm. Additionally, these spectrum profiles exhibited no dependence on the rotation angle of sample substrates with respect to the optical axis of the CD spectrometer. As shown in the spectra of chiral d- and l-isovaline films, the Cotton signs of d- and l-bodies are symmetric, as in the case of d- and l-alanine films, but the signs are inverted from those in the alanine case. Considering configuration of them, it is concluded that the 205 and 175 nm peaks can also be identified as originating from the transitions of carboxyl group (n-π*) and (π-π*), respectively.

**Figure 12.**
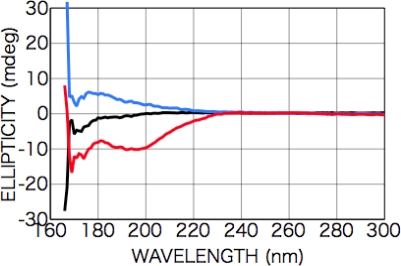
CD spectra of dl-isovaline films as deposited (black) (100-nm in thickness) and after CPL irradiation (colored). Left-handed CPL (blue); right handed CPL (red).

**Figure 13.**
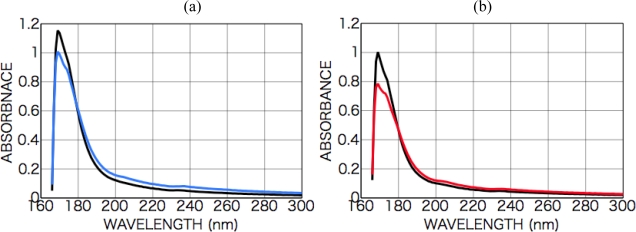
Photo absorption spectra of dl-isovaline films as-deposited (black) and after CPL irradiation (colored). (a) Left-handed CPL (blue); (b) Right handed CPL (red).

After the irradiation with the CPL (wavelength 215 nm, total irradiation energy 2 mWhour), broad peaks appeared in the CD spectra at 175 [carboxyl group (π-π*)] and 200 nm [carboxyl group (n-π*)] with an opposite sign (Figure 12). These results suggest that a chiral structure was introduced into the racemic isovaline film by the CPL. As shown in the absorption spectra (Figure 13), the absorbance after CPL irradiation increased on the low-energy side but decreased on the high-energy side, suggesting some molecular structure change occurred as mentioned above as in the case of phenylalanine and alanine films.

### 2.4. Discussion

To artificially introduce chirality into solid-phase bio-organic molecules, we measured the optical anisotropy in solid-phase films of racemic amino acids after irradiation with asymmetric excitation sources. When we used the aromatic amino acids phenylalanine and tryptophan as the starting materials, two-dimensional anisotropic structure expressed as LD and/or LB was introduced into the racemic films by LPL irradiation. It is suggested that the amino acid molecules in the film were selectively oriented or distorted as the direction of transition dipole polarization becomes oriented so that it corresponds to the LPL direction.

On the other hand, with CPL irradiation, Cotton peaks appeared in the CD spectra at almost the same peak positions as those of corresponding chiral d- or l-amino acid films. The intensities of the Cotton peaks were almost independent of sample angle change, and the signs of the Cotton peaks kept identically corresponding to the rotation direction of the left- or right-handed CPL.

When we used the simple α-hydrogen amino acid alanine and α-methyl amino acid isovaline as the starting materials, the same results as those for phenylalanine were obtained after the irradiation with CPL. With a comparison of the CD spectra of as-deposited chiral films and CPL-irradiated films, these results for CPL irradiation cannot be explained as a simple selective photolysis scheme. For example, 216-nm left-handed CPL is preferentially absorbed by l-alanine with positive Cotton peak in this wavelength region (l-alanine absorbance is larger than that of d-alanine). Supposing that simple l-alanine preferential photolysis occurred, the residual film might show d-alanine-like CD spectra. As shown in the experimental result, however, residual film showed l-alanine-like CD spectra in shorter wavelength region. Similar kinds of results were obtained in phenylalanine and isovaline cases. Whatever the case, by CPL irradiation, chiral structures expressed as CD were successfully introduced into the racemic films.

One speculative explanation is that the preferential photoabsorption and photolysis of surface molecules possibly cause several kinds of structural change, such as structure distortion, conformation change, multimer production, and so on. Because these molecules give different effects for CD spectra and obtained CD spectra should be complicated summation of the effects due to these molecules, accurate understandings of the phenomena and careful experimental treatments are necessary in future work.

Taken together, our experimental results suggest that polarized light sources in space have a relationship with the origin of terrestrial homochirality, working as asymmetric excitation sources that can introduce optical anisotropy and chiral structure into bio-organic molecules or more complex organic compounds.

## 3. Experimental Section

### 3.1. Amino Acid Film Formation

**Figure 14.**
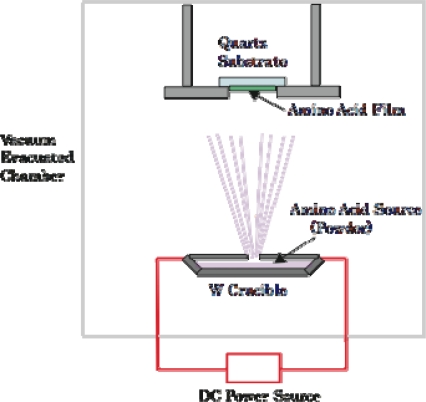
Schematic view of the vacuum evaporation system. Dimension of the chamber is 500 x 500 x 800 mm^3^.

**Figure 15.**
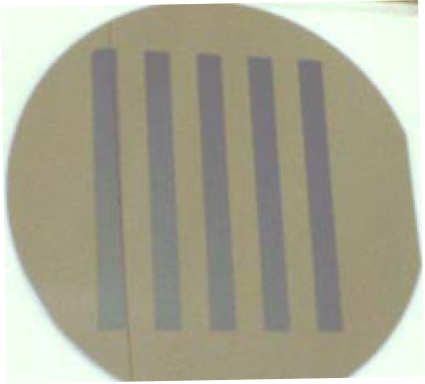
Photograph of dl-phenylalanine film deposited on silicon wafer (100-mm in diameter) through a 5-line pattern stencil mask.

As the sample of solid-phase achiral bioorganic molecules, we formed thin solid films of the racemic amino acids on a CaF_2_ or MgF_2_ substrates from crystal powders of dl-body amino acids using a thermal-crucible vacuum evaporator (ANELVA EVP-38230) (Figure 14). Sublimation temperature was 40 ~ 70°C depending on amino acid species and pressure of the vacuum chamber was approximately 1 ~ 5 x 10^5^ Pa throughout the evaporation process. In a similar way, chiral films were also deposited from d- and l-body powders of the amino acids for comparison. The thickness of the deposited films was in-situ measured with a crystal oscillator mounted in the evaporation chamber. For calibration of the crystal oscillator, the thickness and refractive index were simultaneously determined by using reflection auto-ellipsometry after the deposition of each film. Figure 15 is a photograph of dl-phenylalanine film deposited on a silicon wafer through a 5-line pattern stencil mask. To clarify the optical anisotropy of the amino acid films, we measured the CD spectra of the deposited films using a commercial CD spectrometer (JASCO J-725).

**Figure 16.**
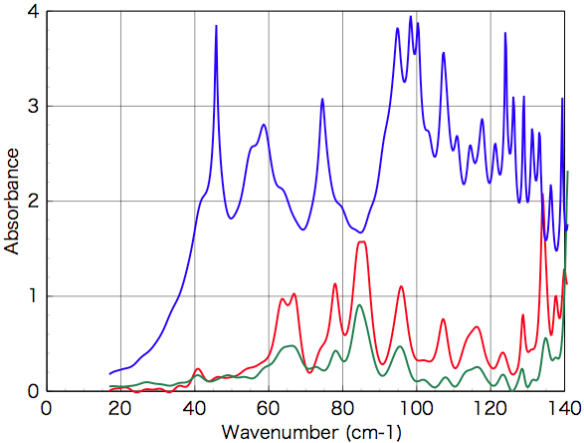
Terahertz absorption spectra of d- (green) and l- (red) and dl-isovaline (blue).

Powders of d-, l-, dl-phenylalanine, d-, l-, dl-tryptophan, d-, l-, dl-alanine, and d-, l-isovaline were purchased from Kanto Chemical Co., Inc. (Tokyo, Japan). dl-isovaline powder was abiotically synthesized in the laboratory of Fukuoka Institute of Technology (Fukuoka, Japan). The optical purity of synthesized racemic dl-isovaline was confirmed using CD spectra of the evaporated films (Figures 11 & 12) and Terahertz absorption spectra of the crystal powders condensed in a polyethylene pellet (Figure 16).

### 3.2. Terahertz Absorption Spectroscopy

The Terahertz absorption spectra in Figure 16 were measured using a Terahertz spectrometer THz-TDS2004 (Aispec) at the sample temperature 77 K. As shown in the figure, the absorption spectra of chiral d- and l-isovaline is similar but the spectrum of the racemic dl-isovaline has a different profile from those of chiral iosovaline. Absorption spectra in these energy region have been advokated to be attributed to weak intermolecular interactions, especially hydrogen bonding between molecules. In this reason, racemic crystals including interactions between heterogeneous isomers (l-d) and chiral crystals including interactions between homogeneous isomers (l-l or d-d) can be clearly distinguished using this method. Yamaguchi *et al.* already reported Terahertz absorption spectra of racemic- and chiral alanine (α-hydrogen amino acid) [26], and our present result is the first observation for α-methyl amino acids.

### 3.3. Optical Anisotropy Measurements of the Amino Acid Solutions and Films

#### 3.3.1. Phenylalanine

As a preliminary step in the measurement, we measured CD spectra of aqueous solution of d-, l-, dl-phenylalanine (pH = 7) using a synthesized-quartz optical cell with 0.1 mm of optical pass length. Peaks having opposite Cotton signs were observed at 215 and 190 nm in the symmetric d- and l-phenylalanine spectra, although the racemic dl-phenylalanine spectrum exhibited no peaks. The 215- and 190-nm Cotton peaks are well-known as those originating from the transition of the carboxyl group (n-π*) and the benzene ring (π-π*), respectively [27]. It must be noted that Nishino *et al.* report that 215-nm Cotton peak should be attributed to not only the carboxyl group (n-π*) also overlapping of the carboxyl group (n-π*) and the benzene ring (π-π*) [28]. As for the as-deposited films before polarized light irradiation, CD spectra of the racemic dl-phenylalanine film exhibited no peaks as shown in Figure 2(b), as was the case for the aqueous solution. Meanwhile, three peaks were observed in the spectra of chiral films deposited from d- or l-phenylalanine at 226, 204, and 188 nm as shown in Figure 2(a). Additionally, these spectrum profiles exhibited no dependence on the rotation angle of sample substrates with respect to the optical axis of the CD spectrometer. As shown in the spectra of chiral d- and l-phenylalanine films, the Cotton signs of d- and l-body are symmetric and the sign of the 204-nm peak are inverted from the 226- and 188-nm peaks. From the comparison with the spectra of aqueous solution of d-, and l-phenylalanine, the 226- and 204-nm peaks of the films are identified as those shifted in the longer wavelength direction from the 215- and 190-nm peaks of the aqueous solution, respectively. It is therefore naturally concluded that the 226- and 204-nm peaks can also be identified as originating from the transition of the carboxyl group (n-π*) and aromatic ring (π-π*), respectively. By the analogical consideration, the 188-nm Cotton peak of observed in film spectra can be estimated as originating from the transition to a higher level of carboxyl group (π-π*), in spite of corresponding Cotton peaks not being observed in the case of aqueous solution. We speculate that the observed Cotton peak shift to the low-energy direction is due to molecular conformation change from the hydrated structure in aqueous solution to anhydrous circumstances in solid film, as in the case of alanine mentioned below.

#### 3.3.2. Tryptophan

For another aromatic amino acid, d-, l-, dl-tryptophan, CD spectra of aqueous solution (pH = 7) and solid film (Figure 5) were also measured. Peaks with opposite Cotton signs were observed at 224 and 194 nm in the symmetric d- and l-phenylalanine spectra. The 224- and 194-nm Cotton peaks are well-known as those originating from the transition of the carboxyl group (n-π*) and indole ring (π-π*), respectively [29]. As for the chiral films deposited from d- or l-tryptophan, similar spectrum profiles were observed with Cotton peaks positioned at 231 and 195 nm. There were a slight shift to the low-energy direction and there was no dependence on the rotation angle of the sample substrates, as in case of phenylalanine as mentioned above. Cotton peaks at 231 and 195 nm can be identified as those originating from the transition of the carboxyl group (n-π*) and indole ring (π-π*), respectively.

#### 3.3.3. Alanine

It has been reported that aqueous solution of l-alanine presents 200-nm Cotton peaks and well-known be identified as those originating from the transition of the carboxyl group (n-π*) [30,31]. As for the as-deposited films before polarized light irradiation, CD spectra of the racemic dl-alanine film exhibited no peaks, but peaks were observed in the spectra of chiral films deposited from d- or l-alanine at 210 and 180 nm (Figure 8). Additionally, these spectrum profiles exhibited no dependence on the rotation angle of sample substrates with respect to the optical axis of CD spectrometer. As shown in the spectra of chiral d- and l-alanine films, the Cotton signs of d- and l-body are symmetric. From the comparison with the spectra of aqueous solution of d-, and l-alanine, the 210- and 180-nm peaks of the films are identified as those shifted to the longer wavelength direction from the 200-nm peak and the shorter wavelength peaks of aqueous solution, respectively. It is, therefore, naturally concluded that the 210- and 180-nm peaks can also be identified as originating from the carboxyl group transitions (n-π*) and (π-π*), respectively. However, in a pioneering experimental work of amino acid films by Tanaka *et al.* [32] and a recent theoretical calculation work by Kaneko *et al.* [33], it is advocated that 210-nm peak of alanine film is derived from surface scattering and real carboxyl group transitions (n-π*) peak is located at 200-nm with opposite Cotton sign. It is sure that our CD spectra also show faint symptoms of opposite sign Cotton peaks at 200-nm. It is supposed that the opposite sign Cotton peaks at 200-nm could not be clearly observed due to the film thickness of our sample. Understanding these situations, we tentatively nominate 210-nm peak for the carboxyl group transitions (n-π*) in this paper.

#### 3.3.4. Isovaline

CD spectra of the racemic dl-isovaline film exhibited no peaks (black line in Figure 12), but peaks were observed in the spectra of chiral films deposited from d- or l-isovaline at 205 and 175 nm as shown in Figure 11. Additionally, these spectrum profiles exhibited no dependence on the rotation angle of sample substrates with respect to the optical axis of the CD spectrometer. As shown in the spectra of chiral d- and l-isovaline films, the Cotton signs of d- and l-body are symmetric as in case of d- and l-alanine films, but the signs are inverted to the alanine case. Considering the configuration of them, it is concluded that the 205- and 175-nm peaks can also be identified as originating from the transition of carboxyl group (n-π*) and (π-π*), respectively.

## 4. Conclusions

For the purpose of experimentally verifying the evidence of the origin of terrestrial homochirality, we measured optical anisotropy in solid-phase amino acids after LPL-SR and CPL-FEL irradiation. After the irradiations, peaks appeared in the CD spectra at almost the same peak positions as those of corresponding non-racemic d- or l-amino acid films. With LPL-SR irradiation, linear anisotropic structures optically expressed as LD and/or LB was introduced into the racemic films. With CPL-FEL irradiation, chiral structures optically expressed as CD were introduced into the racemic film. These results suggest that polarized light sources in space has relationship with the origin of terrestrial homochirality, working as asymmetric excitation sources that can introduce optical anisotropy and chiral structure into bio-organic molecules or more complex organic compounds. The emergence of symmetrical optical anisotropy corresponding to the rotation direction of CPL is effective enough to demonstrate asymmetric reactions on the surfaces of space materials, which will reveal the origins of terrestrial homochirality. The mechanism of the photochemical asymmetric reactions in “soft materials”, such as amino acid films, must be investigated further. On the other hand, why l-amino acid and d-sugar dominant instead of d-amino acid and l-sugar in terrestrial homochirality still remains unexplained. Further experimental and theoretical approaches should be attempted, including consideration of phenomena related to intrinsic symmetry breaking in nature.
